# Traditional uses of medicinal plants to prevent and treat diabetes; an updated review of ethnobotanical studies in Iran

**DOI:** 10.15171/jnp.2017.20

**Published:** 2017-01-15

**Authors:** Majid Asadi-Samani, Mohammad-Taghi Moradi, Leila Mahmoodnia, Shahla Alaei, Fatemeh Asadi-Samani, Tahra Luther

**Affiliations:** ^1^Student Research Committee, Shahrekord University of Medical Sciences, Shahrekord, Iran; ^2^Medical Plants Research Center, Shahrekord University of Medical Sciences, Shahrekord, Iran; ^3^Department of Internal Medicine, Shahrekord University of Medical Sciences, Shahrekord, Iran; ^4^Department of General Surgery, University of Michigan, Ann Arbor, Michigan, USA

**Keywords:** Blood sugar, Traditional medicine, Ethnobotany, Iran

## Abstract

**Background:**

Obesity and physical inactivity are currently on the rise due to industrialization of the communities, which has recently led to increased incidence of different diseases such as diabetes. Epidemiological studies and figures have demonstrated the growing incidence of diabetes. Relevantly, the side effects of chemical drugs have led patients to use medicinal plants and traditional approaches despite advances in development of chemical drugs. The aim of this review article is to report the medicinal plants and their traditional uses to prevent and treat diabetes according to the findings of ethnobotanical studies conducted in different regions of Iran.

**Evidence Acquisitions:**

The search terms including ethnobotany, ethnomedicine, ethnopharmacology, phytopharmacology, phytomedicine, Iran, and traditional medicine in combination with diabetes, blood sugar and hyperglycemic were searched from scientific databases.

**Results:**

The results of this article can be a comprehensive guideline, based on ethnobotany of different regions of Iran, to prevent and treat diabetes. According to this review article, certain plant species such as Urtica dioica L., popularly called nettle, in eight regions, Teucrium polium L., popularly called poleigamander, in five regions, and Trigonella foenum-graecum L., Citrullus colocynthis (L.), Schrad., and Juglans regia L. in four regions, were reported to be frequently used to prevent and treat diabetes

**Conclusions:**

The introduced medicinal plants in this review can be investigated in further research and produce new drugs with limited side effects

**Implication for health policy/practice/research/medical education:**

The introduced medicinal plants in this review paper can be new options, based on ethnobotany of different regions of Iran, to use in subsequent laboratory research and clinical trials for discovering and producing herbal drugs for prevention and treatment of diabetes.

## 1. Context

Diabetes is a metabolic and multifactorial disorder which is characterized by chronically increased levels of blood sugar and develops due to disturbed secretion and/or function of insulin (1,2). From clinical perspective, diabetes mellitus is considered to be one of the most important risk factors for certain disorders such as nephropathy, retinopathy, neuropathy, and cardiovascular disease, and according to projections, its prevalence will be increasing in human communities in the future (3-5).

Different severities of insulin deficiency and resistance are seen in patients with diabetes mellitus, and when these patients are not treated by diet, physical activity, and hypoglycemic drugs, it is necessary to treat them by other approaches. Although use of insulin and hypoglycemic drugs is currently considered to be the main and effective treatment for diabetes mellitus, they may cause different complications such as increased lipid reserves, shrinkage of lipid at injection site, and hypoglycemic shock. In addition, insulin and hypoglycemic drugs affect pathogenesis of debilitating complications due to diabetes (6). Since diabetes mellitus is one of the oxidative stress-associated diseases (7), use of antioxidants can be a therapy approach to control diabetes and reduce associated complications (8-10).

Recently efforts have been increasingly made to use alternative medicine such as herbal drugs. Actually use of medicinal plants has caused a decrease in incidence of different diseases because of their effects in protecting against oxidative damage and decreasing inflammation (11-22). In this regard, recent studies have sought to investigate traditional uses and in vitro effects of medicinal plants, and to identify and isolate their active compound to develop herbal drugs (23-29). Therefore, the aim of this study is to identify medicinal plants and their traditional uses to prevent and treat diabetes according to the findings of the ethnobotanical studies conducted in different regions of Iran to offer some strategies to produce new and more effective herbal drugs to researchers.

## 2. Evidence Acquisition

In this review article, the words ethnobotany, ethnomedicine, ethnopharmacology, phytopharmacology, phytomedicine, Iran, and traditional medicine in combination with diabetes, blood sugar and hyperglycemic were used to retrieve relevant articles from scientific databases. Duplicate articles and the articles without accessible full text were not included in final analysis.

## 3. Results

**Figure 1:**
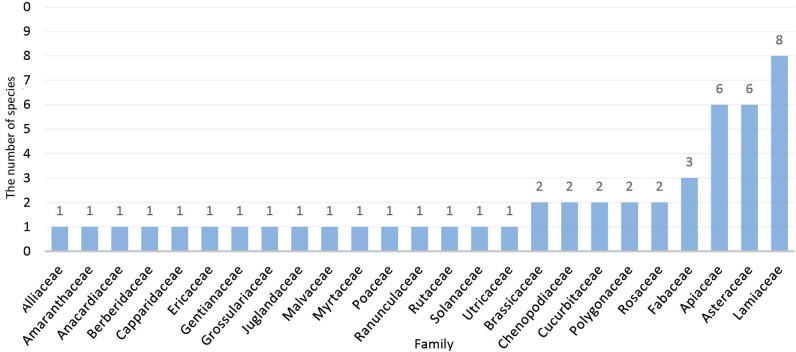
A number of medicinal species effective on diabetes in each family

The present study indicated that Iran’s people of different cultures and in various regions use 49 species of medicinal plants from 25 families based on traditional medicine to specifically to treat blood sugar. Most of the identified plants were from Lamiaceae, Asteraceae and Apiaceae family with eight, six and six species respectively (Figure 1). Table 1 gives further data on the medicinal plants effective on kidney stone disease.

**Table 1:** Medicinal plants effective on diabetes in different subcultures and regions of Iran

**No.**	**Scientific name**	**Family**	**Local name**	**Used organs**	**Preparation**	**Regions**	**Ref.**
1	*Centaurea bruguieriana* Hand. Mzt.	Asteraceae	Badverd, Bad Bord	Leaves, flowers	Infusion	Northeast Latrine Zone of Persian Gulf and Arjan, Fars Province	(30,31)
2	*Citrullus colocynthis* (L.) Schrad.	Cucurbitaceae	Gorjey-e Abu jahl, khiar gorgu, Hendevaney e sangi	Seeds	Powdered ripen seeds	Kohghiluyeh va Boyer Ahmad province; Northeast Latrine Zone of Persian Gulf; Khuzestan province and Arjan, Fars province	(30-33)
3	*Otostegia persica* (Burm.) Boiss.	Lamiaceae	Shekar Shafa	Fruit	Decoction	Arjan, Fars province and Mobarakeh, Isfahan province	(31,34)
4	*Teucrium polium* L.	Lamiaceae	Alpe, Chez Koohi, Kalporeh	Aerial parts	Infusion, Decoction	Arjan, Fars province; Chaharmahal va Bakhtiyary province; Mashhad, Razavi Khorasan province; Northeast Latrine Zone of Persian Gulf and Khuzestan province	(30-32,35,36)
5	*Coriandrum sativum* L.	Apiaceae	Gishniz	Leaves, Seed, stems	Edible, Decoction	Arjan, Fars province and Mobarakeh region, Isfahan province	(31,34)
6	*Capparis spinosa* L.	Capparidaceae	Kewerak-Lagjin	Stems, fruit, flowers, leaves, roots, seed	Edible	Arjan, Fars province; Turkmen Sahra, north of Iran and Northeast Latrine Zone of Persian Gulf	(30,31,37)
7	*Tanacetum polycephalum* (L.) Schultz-Bip.	Asteraceae	Mokhaleseh	Leaves, flowers	internal	Chaharmahal va Bakhtiyary province	(35)
8	*Urtica dioica* L.	Utricaceae	Gazane, Chitchiti odghin, Gazgazuk	Root, aerial parts	Decoction, Infusion	Hezar Mountain, South East of Iran; Khuzestan province; Maraveh Tappeh Region, North of Iran; Kohghiluyeh va Boyer Ahmad province; Turkmen Sahra, north of Iran; Zarivar, Kordestan province; Chaharmahal va Bakhtiyary province and Northeast Latrine Zone of Persian Gulf	(30,32,33,35,37-40)
9	*Levisticum officinale* W. D. Koch.	Apiaceae	Karafse kuhi	Leaf, root, seed	Infusion, Flavoring with yogurt, use as vegetable	Hezar Mountain, South East of Iran	(38)
10	*Arctium lappa* L.	Asteraceae	Baba adam	Root, leaves	Decoction, Oral	Hezar Mountain, South East of Iran and Khuzestan province	(32,38)
11	*Cichorium intybus* L.	Asteraceae	Kasni, Kashni	Whole plant	Orally, cooked and taken with yogurt, powdered flower, Arrack, Decoction	Hezar Mountain, South East of Iran and Kohghiluyeh va Boyer Ahmad province	(33,38)
12	*Berberis integerrima* Hort. ex K.Koch.	Berberidaceae	Zereshk	Flower, fruit, root	Decoction,	Kohghiluyeh va Boyer Ahmad province and Hezar Mountain, South East of Iran	(33,38)
13	*Juglans regia* L.	Juglandaceae	Gerdoo, Ghoz	Leaves, bulb, fruit, root	Infusion, Aromatic water and powder, Edible	Hezar Mountain, South East of Iran; Sirjan, Kerman province; Mobarakeh region, Isfahan province and Zarivar, Kordestan province	(34,38,40,41)
14	*Centaurium tenuifolium* (Hoffm. & Link) Fritsch	Gentianaceae	Ghontorion	Flower, leaves	Brew fresh organ	Hormozgan province	(42)
15	*Bienteria cycloptera* Bunge	Chenopodiaceae	Andere, samsil, samsul	Leaves	Decoction	Hormozgan province	(42)
16	*Salvia mirzayanii* Rech. F. & Esfand	Lamiaceae	Moortalkh, marve tahl, shir ghanam, mor porzu	Leaves	Powder decoction	Hormozgan province	(42)
17	*Haussknechtia elymaitica* Boiss.	Apiaceae	Kelos-e kuhi	Aerial part	Orally	Kohghiluyeh va Boyer Ahmad province	(33)
18	*Achillea wilhelmsii* K.Koch.	Asteraceae	Berenjas	Flower	Powder and infusion of dried flowers	Kohghiluyeh va Boyer Ahmad province	(33)
19	*Astragalus fasciculifolius* Boiss.	Fabaceae	Gineh, Ginja	Flower, root, gum	Decoction	Kohghiluyeh va Boyer Ahmad province	(33)
20	*Trigonella foenum graecum* L.	Fabaceae	Shanbalileh	Aerial part, seed	Infusion	Kohghiluyeh va Boyer Ahmad province; Mobarakeh region, Isfahan province; Mashhad, Razavi Khorasan province and Khuzestan province	(32-34,36)
21	*Thymus daenensis* ˇCelak.	Lamiaceae	Halpeh	Aerial part	infusion, extract of fresh plant,	Kohghiluyeh va Boyer Ahmad province	(33)
22	*Dysphania botrys* (L.) Mosyakin & Clemants	Amaranthaceae	Dermaneh Torki	Aerial parts	-	Mashhad, Razavi Khorasan province	(36)
23	*Rhus coriaria* L*.*	Anacardiaceae	Somagh	Fruit	-	Mashhad, Razavi Khorasan province	(36)
24	*Nasturtium officinale* R. Br.	Brassicaceae	Alafe cheshmeh, bulag ote	Aerial parts	infusion	Mashhad, Razavi Khorasan province and West Azerbaijan province	(36,43)
25	*Vaccinium arctostaphylos* L.	Ericaceae	Ghareh Ghat	Fruit	-	Mashhad, Razavi Khorasan province	(36)
26	*Securigera securidaca* (L*.*) Degen & Dorfl.	Fabaceae	Gandeh Talkheh	Seed	-	Mashhad, Razavi Khorasan province and Khuzestan province	(32,36)
27	*Ribes khorasanicum* Saghafi & Assadi	Grossulariaceae	Ghareh Ghat	Fruit	-	Mashhad, Razavi Khorasan province	(36)
28	*Salvia leriifolia* Benth.	Lamiaceae	Noruzak	Aerial parts	-	Mashhad, Razavi Khorasan province	(36)
29	*Syzygium aromaticum* (L.) Merr. & L.M.Perry	Myrtaceae	Mikhak	Flower	-	Mashhad, Razavi Khorasan province	(36)
30	*Polygonum aviculare* L*.*	Polygonaceae	Alaf Haftband	Aerial parts	-	Mashhad, Razavi Khorasan province	(36)
31	*Rheum turkestanicum* Janisch.	Polygonaceae	Eshghan	Root	-	Mashhad, Razavi Khorasan province	(36)
32	*Toddalia asiatica* (L.) Lam*.*	Rutaceae	Dahan baz-Dahan basteh	Fruit		Mashhad, Razavi Khorasan province	(36)
33	*Mentha spicata* L.	Lamiaceae	Nana	Aerial parts, leaves, essence	-	Mobarakeh region, Isfahan province	(34)
34	*Allium cepa* L.	Alliaceae	Sir	Bulb, Aerial parts	-	Mobarakeh region, Isfahan province	(34)
35	*Cucurbita pepo* Mill.	Cucurbitaceae	Kado halvai	Seed, fruit	-	Mobarakeh region, Isfahan province	(34)
36	*Petroselinum crispum* Mill.	Apiaceae	Jafari	Root, leaves fruit, Essence	-	Mobarakeh region, Isfahan province	(34)
37	*Hordeum vulgare* L*.*	Poaceae	Jo dosar	Seed, bran	-	Mobarakeh region, Isfahan province and Sardasht, Western Azerbaijan	(34,44)
38	*Althaea officinalis L.*	Malvaceae	Hero	Flowers, roots, leaves	-	Sardasht, Western Azerbaijan province	(44)
39	*Anethum graveolens* L.(dill)	Apiaceae	Toragh	Leaves	-	Sardasht, Western Azerbaijan	(44)
40	*Brassica Napus L.*	Brassicaceae	Kolza	Root, seed, leaves	-	Sardasht, Western Azerbaijan and Khuzestan province	(44)
41	*Lamium album L.*	Lamiaceae	Gaz gaz	Leaves	-	Sardasht, Western Azerbaijan	(44)
42	*Suaeda altissima* Pall.	Chenopodiaceae	mangak	Leaves, stems	-	Northeast Latrine Zone of Persian Gulf	(30)
43	*Helianthus tuberosus* L.	Asteraceae	-	Tuber and leaves	Decoction	Zarivar, Kordestan province	(40)
44	*Kelussia odoratissima* Mozaff.	Apiaceae	Keluss	Whole plant	-	Khuzestan province	(32)
45	*Salvia officinalis* L.	Lamiaceae	Chilaver	Leaves, fruit	-	Khuzestan province	(32)
46	*Ranunculu sarvensis* L.	Ranunculaceae	Zard gad	Flowers	-	Khuzestan province	(32)
47	*Amygdalus scoparia* Spach.	Rosaceae	Baym	Gum	-	Khuzestan province	(32)
48	*Cerasus mahaleb* L.	Rosaceae	Mahlou	Fruit	-	Khuzestan province	(32)
49	*Solanum nigrum* L.	Solanaceae	Hava	Aerial parts, fruit	-	Khuzestan province	(32)

According to the findings, some of the plant species are used to treat diabetes in more than one region. For example, *Urtica dioica* L., popularly called nettle, is used in eight regions, *Teucrium polium* L., commonly called poleigamander, in five regions, *Trigonella foenum graecum* L., *Citrullus colocynthis* (L.) Schrad., and *Juglans regia* L., in four regions, and *Capparis spinosa* in three regions are used to treat diabetes (Table 1; 30-44).

Controlling glycemia completely by use of chemical drugs without causing the complications due to these drugs remains the concern in health-related studies and practice. It seems that other approaches should be sought out to treat hypoglycemia which is known as a hidden epidemic. Traditional medicine and use of medicinal plants is a supplementary and auxiliary method which has offered an effective approach to prevent and treat diseases (45-52).

It is believed that oxidative stress contributes to development of vascular complications in patients with diabetes. Increased reactive oxygen species (ROS) levels in diabetes may be due to decreased destruction and/or increased production of catalase (CAT--enzymatic/non-enzymatic), superoxide dismutase (SOD) and glutathione peroxidase (GSH–Px) antioxidants. The variations in the levels of these enzymes cause the tissues to become susceptible to oxidative stress, leading to development of diabetic complications (53). The results of this review indicated the majority of the reported plants are from families Lamiaceae (eight species), Asteraceae and Apiaceae (six species) that have high concentrations of phenolic compounds (54,55). Anti-diabetic effects of phenolic compounds have already been confirmed (56,57). Phenolic compounds may have a protective effect against hyperglycemia-induced chronic diseases through both protection of antioxidants and inhibition of starch digestion. Co-application of phenolic compounds and synthetic enzyme inhibitors may decrease the effective dose of synthetic enzyme inhibitors that are needed to control postprandial glycemia (56).

## 4. Conclusions

The present study indicated that Iranian people from different cultures and in different regions consume 49 species of medicinal plants from 25 families based on traditional medicine to treat hyperglycemia. This demonstrates that Iran’s traditional medicine is rich. Iran’s traditional medicine has long addressed use of nature-based resources to prevent and treat diabetes. The availability of various approaches to use plants to treat diseases, including hyperglycemia, in Iranian traditional medicine conforms to geographical and vegetational conditions of this country. The findings of this study can be a comprehensive guideline, based on ethnobotany of different regions of Iran, to prevent and treat diabetes.

## Authors’ contribution

MTM, SA, LM, and FAS searched the databases. MAS, MTM, SA, and FAS wrote the draft. MAS and TL edited the manuscript. All authors read and approved the final version.

## Conflicts of interest

There is not conflicts of interest to declare.

## Funding/Support

None.
